# An Optimized Protocol for the Differentiation and Fibrosis-Associated Transcriptional Priming of THP-1 Macrophages

**DOI:** 10.3390/mps9050140

**Published:** 2026-09-25

**Authors:** Janya Khattiya, Chareeporn Akekawatchai

**Affiliations:** 1 Graduate Program in Biomedical Sciences, Faculty of Allied Health Sciences, Thammasat University, Pathumthani 12121, Thailand; 2 Research Unit in Diagnostic Molecular Biology of Chronic Diseases Related to Cancer (DMB-CDC), Department of Medical Technology, Faculty of Allied Health Sciences, Thammasat University, Pathumthani 12121, Thailand; cakekawatchai@gmail.com or ejareepo@tu.ac.th

**Keywords:** THP-1 cells, phorbol 12-myristate 13-acetate (PMA), macrophage differentiation, CXCL12, *TGF-β*, fibrogenesis, in vitro model

## Abstract

Monocyte-to-macrophage differentiation and subsequent activation play a crucial role in tissue fibrosis. While the human monocytic cell line THP-1 is widely used as an in vitro model, variations in the use of phorbol 12-myristate 13-acetate (PMA) differentiation often led to inconsistent macrophage phenotypes. Here, we present an optimized protocol yielding mature differentiated THP-1 macrophages (d-THP-1) with stable baseline competence for fibrosis-associated transcriptional change. A protocol consisting of 48 h PMA treatment followed by a 24 h resting phase successfully generated macrophage-like THP-1 cells, confirmed by microscopic assessment and flow cytometric analysis of surface markers. Furthermore, the responsiveness of these optimized macrophages to the chemokine CXCL12 was validated. Following stimulation, the optimized cells exhibited a potentially fibrosis-associated state, characterized by significant early upregulation of *matrix metalloproteinase 9 (MMP9)* and late upregulation of *transforming growth factor-β (TGF-β)* measured by RT-qPCR. This reproducible protocol provides a reliable in vitro platform for studying macrophage-mediated fibrotic pathways and screening of therapeutic targets.

## 1. Introduction

Macrophages are key orchestrators of the inflammatory and fibroproliferative responses following tissue injury. Upon chronic activation, they undergo pro-fibrogenic polarization and secrete key pro-fibrotic cytokines, notably transforming growth factor-β (*TGF-β*). This sustained signaling drives myofibroblast differentiation and excessive extracellular matrix deposition, leading to pathogenic fibrogenesis [1].

The human THP-1 leukemia cell line is a well-established model for studying monocyte/macrophage biology due to its human origin and ease of maintenance compared with primary human monocyte-derived macrophages (MDMs) [2]. Differentiation of THP-1 monocytes into adherent, macrophage-like cells is typically achieved using phorbol 12-myristate 13-acetate (PMA). However, published protocols vary widely in PMA concentration (ranging from 5 to 200 nM) and incubation or resting times [3,4,5], which often induce undesirable baseline activation of inflammatory genes [4,6], high cytotoxicity [6], or inconsistent phenotypic responses [2,4].

Building upon in vitro screening methodologies [3,4,7,8], this protocol details an optimized, low-toxicity PMA differentiation scheme, coupled with an essential resting period (serum starvation). The latter step is incorporated to ensure baseline stabilization prior to downstream macrophage activation. Moreover, to validate the functional competence of these optimized cells, we utilize the CXCL12/CXCR4 signaling axis, which is implicated in driving monocyte- or macrophage-mediated fibrosis in multiple organ systems [9,10,11,12]. In these optimized cells, CXCL12 successfully upregulated *MMP9* and *TGF-β1* expression, suggesting a potentially fibrosis-associated transcriptional response. This system serves as a baseline platform for investigating chemokine-mediated fibrosis and a screening platform for anti-fibrotic drug discovery.

## 2. Experimental Design

The protocol comprises five sequential modules designed to streamline cell differentiation and evaluate functional outcomes: (1) monocyte culture expansion, (2) optimization of PMA-induced THP-1 differentiation, (3) screening and quantification of activation markers in macrophage-like THP-1 cells, (4) CXCL12 stimulation, and (5) downstream RT-qPCR quantification for *TNF-α*, *IL-1β*, *CD163*, *CD206*, *MMP9* and *TGF-β1* expression. To ensure validation, a vehicle-treated control (lacking CXCL12) must run in parallel.

Protocol Limitations: Cells beyond passage 15 exhibit altered differentiation mechanics and must not be used. Furthermore, because serum components can naturally harbor endogenous gene expression, users must strictly transition to serum-starved medium during the chemokine induction phase to prevent background interference and ensure accurate baseline readouts.

### 2.1. Materials

- Human acute monocytic leukemia cell line, THP-1 (ATCC; Cat. no. TIB-202);
- RPMI-1640 medium (Gibco, Grand Island, NY, USA; Cat. no. 11875093);
- Phosphate-buffered saline (PBS), 1× (Cytiva HyClone, Logan, UT, USA; Cat. no. SH30256.02);
- 0.25% trypsin-EDTA (Gibco, NY, USA; Cat. no. 25200072);
- Fetal bovine serum (FBS), qualified (Gibco, NY, USA; Cat. no. 10270-098);
- HEPES, 1M (Gibco, NY, USA; Cat. no. 15630080);
- Sodium pyruvate solution, 100 mM (Gibco, NY, USA; Cat. no. 11360070);
- Penicillin/streptomycin, 100X (Gibco, NY, USA; Cat. no. 15140122);
- Phorbol 12-myristate 13-acetate (PMA) (Sigma-Aldrich, St. Louis, MO, USA; Cat. no. P1585);
- PE-conjugated anti-human CD68 (KP1) mouse monoclonal IgG1 (Santa Cruz, Biotechnology Inc., Santa Cruz, CA, USA, Cat. no. SC-20060);
- PE-conjugated mouse IgG1 (Santa Cruz, Biotechnology Inc., Cat. no. SC-2866);
- FITC-conjugated anti-human *CD163* (GHI/61) mouse monoclonal IgG1 (Santa Cruz, Biotechnology Inc., Cat. no. SC-20066);
- FITC-conjugated mouse IgG1 (Santa Cruz, Biotechnology Inc., Cat. no. SC-2855);
- Paraformaldehyde (PFA) (Sigma-Aldrich, St. Louis, MO, USA; Cat. no. 30525-89-4);
- Recombinant human CXCL12/SDF-1α protein (PeproTech, Cranbury, NJ, USA; Cat. no. 300-28A);
- NucleoSpin^®^ RNA extraction kit (Macherey-Nagel, Düren, Germany; Cat. no. 740955);
- β-Mercaptoethanol (Gibco, NY, USA; Cat. no. 21985023);
- iScript™ Reverse Transcription Supermix (Bio-Rad Laboratories, Hercules, CA, USA; Cat. no. 170-8841);
- iTaq™ Universal SYBR Green Supermix (Bio-Rad Laboratories, Hercules, CA, USA; Cat. no. 172-5121);
- Specific qPCR primers (forward and reverse) for *GAPDH*, *TNF-α*, *IL-1β*, *CD163*, *CD206*, *MMP9* and *TGF-β1* (Integrated DNA Technologies, Coralville, IA, USA).

### 2.2. Equipment

- Six-well flat-bottomed cell culture-treated plate, Costar^®^ TC-treated multiple well plates (Corning Life Sciences, Corning, NY, USA; Cat. no. 3516);
- T-75 (75 cm^2^) cell culture flasks (Corning Life Sciences, NY, USA; Cat. no. 430641U);
- Standard humidified 37 °C, 5% CO_2_ incubator (NuAire Inc., Plymouth, MN, USA; Model no. NU-5710E);
- Biosafety cabinet class II (ESCO Lifesciences, Bangkok, Thailand; Cert. Report no. 0231818);
- Olympus IX50 Inverted microscope;
- Rotofix 32A and Universal 320 R Benchtop centrifuges (Andreas Hettich, Tuttlingen, Germany);
- BD FACS Lyric™ (BD Biosciences, San Jose, CA, USA);
- FlowJo™ software, version 10.8.1;
- NanoDrop™ ONE/ONE^C^ spectrophotometer (Thermo Fisher Scientific, Wilmington, DE, USA);
- T100 Thermal Cycler (Bio-Rad Laboratories, Hercules, CA, USA);
- CFX96 Touch Real-Time PCR Detection System (Bio-Rad Laboratories, Hercules, CA, USA).

## 3. Procedure

### 3.1. Thawing and Culturing of THP-1 Monocytes (Time to Complete: 4–6 Days)

(i) Thaw a vial of THP-1 cells in a 37 °C water bath for 2 min or until the vial thaws approximately 80% of its contents. Resuspend cells in a sterile 50 mL centrifuge tube containing 9 mL of complete RPMI medium (RPMI-1640 supplemented with 10% FBS, 1% Penicillin–Streptomycin, 5 mM HEPES and 1 mM Sodium pyruvate). Centrifuge at 1500 rpm for 5 min. Aspirate the supernatant, gently resuspend the cell pellet in 1–2 mL of complete RPMI medium by pipetting up and down, adjust the final volume to 20 mL with additional complete RPMI medium, and transfer the cell suspension to a T-75 flask.
(ii) Incubate the cells in a humidified 5% CO_2_ incubator at 37 °C. Monitor and maintain the cell density between 2 × 10^5^ and 1 × 10^6^ cells/mL by performing cell counts with a hemocytometer and adding 5–10 mL of fresh complete RPMI-1640 medium to the existing culture every 2–3 days. If the culture volume reaches the maximum working capacity of the vessel or the cell density exceeds 1 × 10^6^ cells/mL, split the cell suspension into the new flasks with fresh medium to re-establish the starting density of 2 × 10^5^ cells/mL.

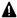
 CRITICAL STEP: Maintain the cell density strictly between 2 × 105 and 1 × 106 cells/mL. Allowing cells to overgrow can lead to spontaneous differentiation or cell death. Subculture THP-1 at least three to four cycles until the cells grow as a single cell suspension before use in any experiments. High passage numbers (>15–20) can reduce the differentiation efficiency of THP-1 cells.

### 3.2. Optimization of PMA-Induced THP-1 Differentiation and the Resting Period (Time to Complete: 3 Days)

An overview of the experimental workflow, including the optimized THP-1 differentiation and resting times, is shown in Figure 1.

- Harvest THP-1 suspension monocytes via centrifugation at 1500 rpm for 5 min. Resuspend in fresh complete RPMI medium.
- Dilute stock PMA with complete RPMI medium to a 2 μg/mL working solution.
- Add the PMA to 6-well tissue culture plates containing 1 mL of complete RPMI medium to achieve final concentrations of 5, 15, and 45 ng/mL for dose optimization. Include wells as untreated or undifferentiated control.
- Seed 1 mL of the harvested THP-1 suspension monocytes into the designated wells at a density of 1 × 10^6^ cells/well in a total volume of 2 mL.
- Incubate plates for exactly 48 h [4] at 37 °C in 5% CO_2_ to promote adherence.
- After the incubation, carefully collect the PMA-containing medium (containing non-adherent cells) into a centrifuge tube. Gently rinse the attached cell monolayer twice with 1 mL of room-temperature 1× PBS and combine the PBS wash fraction with the collected medium.
- Add 1 mL of resting medium (RPMI-1640 without FBS) to each well and incubate for an additional 24 h [4].

**Figure 1:**
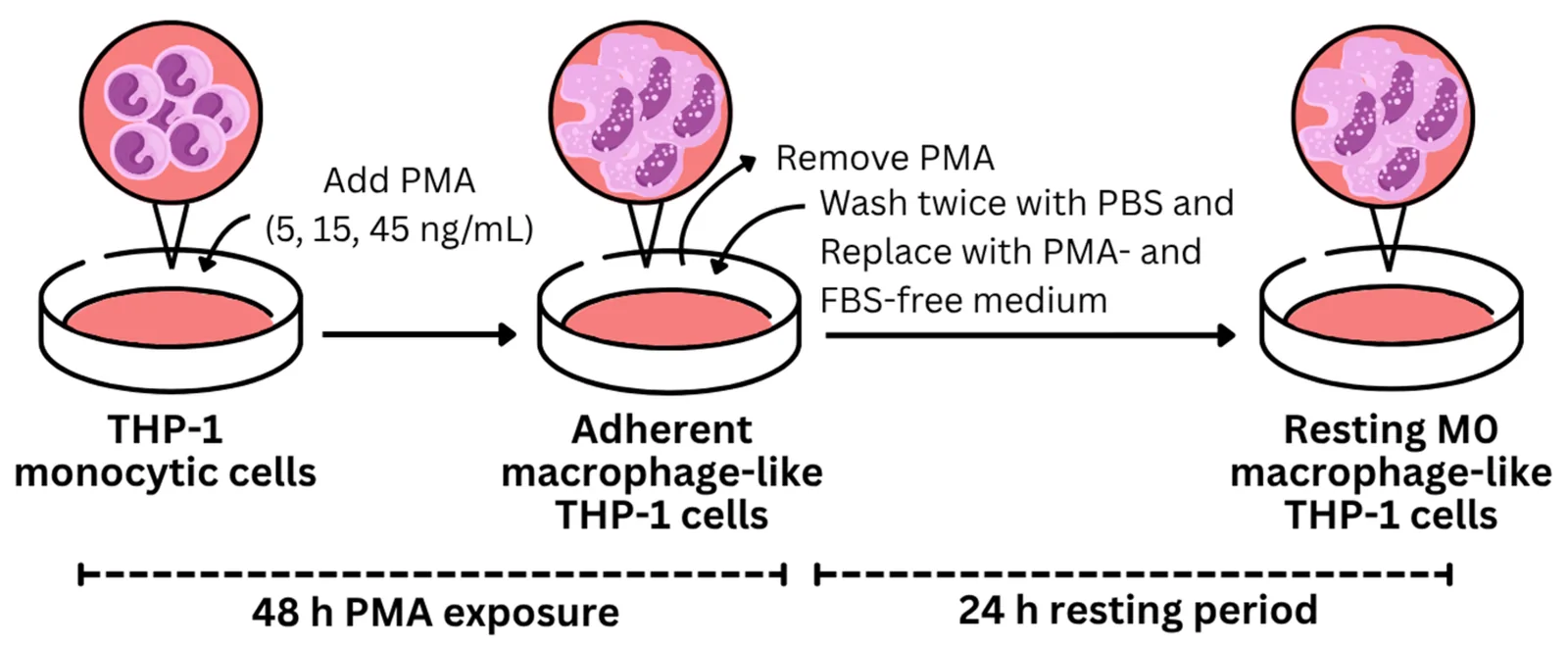
Schematic workflow of the protocol for generating macrophage-like THP-1 cells.


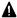
 CRITICAL STEP: Do not pipette PBS directly onto the cell monolayer, as this can induce the detachment of weakly adherent differentiated cells.

viii. Evaluate cell differentiation efficiency and viability as follows:
  - Morphological observation: Observe cell differentiation directly through the 6-well culture plates using an inverted phase-contrast microscope, noting the morphological change from non-adherent (suspension) cells, characterized by a small, round spherical morphology, to adherent cells, identified by spread, enlarged, spindle and irregular shapes anchored to the plate bottom;
  - Quantification of cell adherence via trypan blue exclusion of non-adherent cells: centrifuge the collected supernatant and PBS wash fractions (from Step vi) to pellet non-adherent cells. Resuspend the cell pellet in 1× PBS. Mix the cell suspension 1:1 with 0.4% Trypan blue solution, load onto a hemocytometer, and count viable and non-viable cells under a light microscope.
ix. To determine the optimal PMA concentration, the target benchmark was defined as yielding >90% cellular adherence (assessed by non-adherent cell depletion) while maintaining a high cell viability >80–90%. Note that physical adherence does not directly reflect a 100% phenotypic differentiation efficiency.

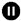
 PAUSE STEP: Following this 24 h resting window, cells enter a stable, non-activated baseline state (M0). Following morphological verification, the plates can be safely maintained for subsequent induction.

### 3.3. Screening and Quantification of Activation Markers in Macrophage-like THP-1 Cells

An overview of the screening and quantification process of activation markers in macrophage-like THP-1 cells is shown in Figure 2. Macrophage differentiation is assessed using immunostaining and flow cytometry, characterized by an increasing forward scatter (FSC) and side scatter (SSC), reflecting an enhanced cell size and granularity, and by expression of the pan-macrophage marker CD68 relative to undifferentiated monocytes.

- Harvest the THP-1 monocytic cells (untreated controls) by centrifugation at 1500 rpm for 5 min and detach the macrophage-like THP-1 cells by trypsinization. Wash the cells once with 1× PBS buffer by centrifugation at 1500 rpm for 5 min. Discard the supernatant and resuspend the cell pellets in 1× human staining buffer (HSB).
- Divide cells into two aliquots. Retain the first aliquot as unfixed (for CD68 staining). Fix the second aliquot (for *CD163* staining) by incubating in 100 μL of 4% PFA in 1× PBS for 15 min at room temperature.
- Centrifuge the fixed cells at 1500 rpm for 5 min to remove the 4% PFA solution. Wash the cells twice with 500 μL of 1× HSB under the same centrifugation conditions. Discard the supernatant, resuspend and adjust the cell concentration of both unfixed and fixed aliquots to 1–5 × 10^6^ cells/mL in 1× HSB.
- Aliquot 50 μL of the cell suspension into 1.5 mL microcentrifuge tubes.
- Add 10 μL of fluorochrome-conjugated test Abs or isotype-matched controls. Incubate on ice for 30 min, protected from light.
- Wash the cells sequentially with 1× HSB, followed by 1× PBS. Resuspend in 1% PFA in 1× PBS for fixation before analysis.
- Acquire data using a BD FACS Lyric flow cytometer. Conduct data analysis using FlowJo software (version 10.8.1).

**Figure 2:**
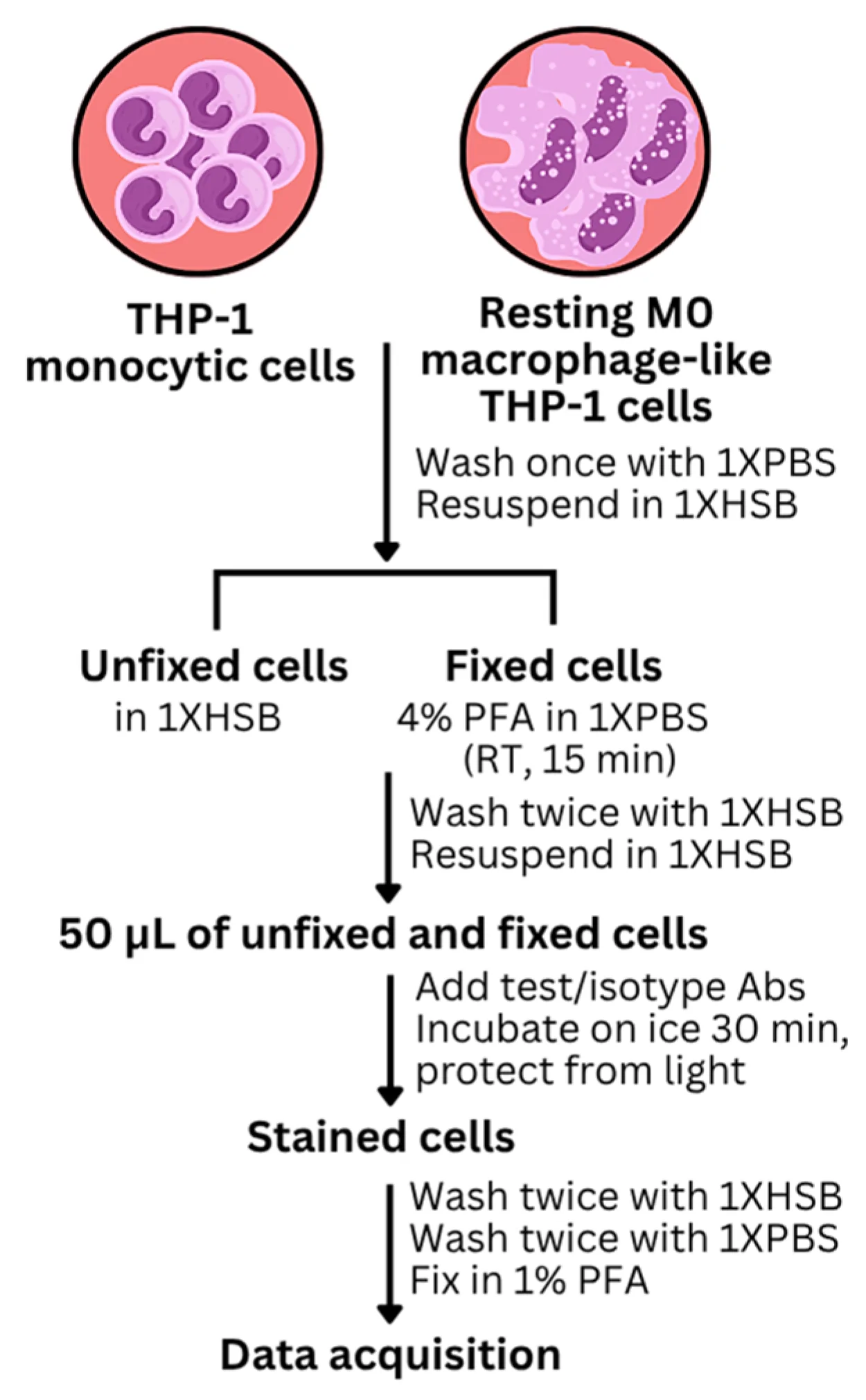
Workflow for surface marker analysis in differentiated macrophage-like THP-1 cells.

### 3.4. CXCL12 Stimulation in THP-1 Cells (Time to Complete: 1–24 h)

An overview of the assay evaluating the fibrosis-associated expression potential of monocytes and M0 macrophage-like THP-1 cells is presented in Figure 3. THP-1 monocytes and resting (M0) macrophage-like cells or differentiated THP-1 (d-THP-1) were stimulated with recombinant human CXCL12 at final concentrations of 10 or 100 ng/mL. Subsequently, an RT-qPCR analysis was performed to quantify specific gene expression.

- Evaluating monocytes in response to CXCL12: seed the THP-1 suspension monocytes into 6-well tissue culture plates at a density of 1 × 10^6^ cells/well.
- Dilute recombinant human CXCL12 stock solution with RPMI-1640 supplemented with 0.1% FBS to prepare a 10 μg/mL working solution.
- Add the CXCL12 working solution to designated wells to achieve final concentrations of 10 and 100 ng/mL (1 and 10 μL, respectively). For the vehicle control, add 10 μL of RPMI-1640 supplemented with 0.1% FBS. Additionally, treat positive control wells with 45 ng/mL PMA in RPMI-1640 supplemented with 10% FBS to confirm maximal gene expression responsiveness [13].
- Evaluating M0 macrophage-like cells in response to CXCL12: Add CXCL12 to the resting M0 macrophage-like THP-1 cells to achieve final concentrations of 10 and 100 ng/mL. The vehicle control was identical to that used in the previous experiment.
- Incubate for 1, 3, and 24 h for downstream RT-qPCR kinetic analysis.

### 3.5. Quantification of Gene Expression by RT-qPCR (Time to Complete: 1–2 Days)

- At indicated times, harvest the cells for RT-qPCR: harvest the suspended monocytes by centrifugation (for monocytes treated with CXCL12) and trypsinize the attached macrophages (for macrophage-like cells treated with CXCL12) into 1.5 mL microcentrifuge tubes.
- Wash the pellets with 1× PBS by centrifugation at 1500 rpm for 5 min.
- Discard the supernatant, leaving the cell pellet in a minimal volume of 1× PBS.

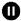
 PAUSE STEP: cell pellets can be stored at −20 °C for up to 1 month prior to RT-qPCR analysis of gene expression.

iv. Perform gene expression; start with cell lysis: lyse the cell pellets collected from the previous step using the lysis buffer provided in the NucleoSpin^®^ RNA extraction kit.
v. Isolate total RNA following the manufacturer’s spin-column protocol.
vi. Quantify RNA purity and concentration using a NanoDrop™ spectrophotometer.
vii. Prepare the iScript Reverse Transcription Supermix according to the manufacturer’s protocol to synthesize cDNA from the template RNA.
viii. Perform reverse transcription in the T100 Thermal Cycler (Bio-Rad Laboratories, Hercules, CA, USA) using the thermocycling conditions listed in Table 1.
ix. Prepare real-time PCR (qPCR) reactions using iTaq Universal SYBR Green Supermix and the primers listed in Table 2, following the manufacturer’s protocol.
x. Perform the reactions in the CFX96 Touch Real-Time PCR detection system using the cycling conditions listed in Table 3.

**Table 1:** Thermal cycling conditions for cDNA synthesis.

Step	Time	Temperature (°C)
Priming	5 min	25
Reverse transcription (RT)	30 min	42
RT inactivation	5 min	85
Cooling the mixture	5 min	4

**Table 2:** Oligonucleotide primer sequences used for real-time PCR validation.

Target Gene	Forward Primer Sequence (5′ → 3′)	Reverse Primer Sequence (5′ → 3′)
*GAPDH* [14]	GGGCATCCTGGGCTACACTG	GAGGTCCACCACCCTGTTGC
*TGF-β1* [15]	CCCTGGACACCAACTATTGC	GTCCAGGCTCCAAATGTAGG
*MMP9* [16]	GCCCCCCTTGCATAAGGA	CAGGGCGAGGACCATAGAG
*CD163* [17]	TTTGTCAACTTGAGTCCCTTCAC	TCCCGCTACACTTGTTTTCAC
*CD206* [18]	TCCGGGTGCTGTTCTCCTA	CCAGTCTGTTTTTGATGGCACT
*IL-1β* [19]	ATGATGGCTTATTACAGTGGCAA	GTCGGAGATTCGTAGCTGGA
*TNF-α* [20]	TCCTTCAGACACCCTCAACC	CACATTCCTGAATCCCAGGT

**Table 3:** Thermal cycling conditions for real-time PCR amplification.

Step	Time	Temperature (°C)
Initial denaturation	5 min	95
Followed by 40 cycles of		
- Denaturation	5 s	95
- Annealing	30 s	60
- Extension	30 s	60
A melting curve analysis from 65 °C to 95 °C with 0.5 °C increments		

## 4. Expected Results

A model for the fibrosis-associated transcriptional priming of monocyte to macrophage-like THP-1 cells has been established. To optimize differentiation conditions, a range of PMA concentrations was evaluated using a 48 h PMA exposure followed by a 24 h resting period. After the PMA exposure and resting phase, PMA-treated THP-1 cells were assessed via phase-contrast microscopy (Figure 4a). In contrast to the non-adherent, spherical untreated controls, cells exposed to escalating doses of PMA (5 to 45 ng/mL) exhibited a distinct, dose-dependent shift toward a macrophage-like lineage (Figure 4a). Cells progressively developed adhesive properties, characterized by marked cytoplasmic spreading, cell flattening, and pseudopodia extensions. Cell clustering and macrophage-like morphological features were most pronounced at PMA concentrations of 15 and 45 ng/mL. To assess cell viability and phenotypic shifts, microscopic evaluation, trypan blue exclusion and high-throughput flow cytometry were performed (Figure 4b–d). The percentage of adherent cells (Figure 4d) is calculated indirectly by subtracting the total number of non-adherent cells from the initial seeding density (1 × 10^6^ cells/well), expressed as a percentage of the initial seeding number. The overall cell viability of the non-adherent fraction is simultaneously determined via the trypan blue exclusion method. While treatment with 5 ng/mL PMA resulted in incomplete monocyte adherence, stimulation with 15 and 45 ng/mL PMA achieved robust cell attachment, exceeding 80% confluency, as evidenced by the depletion of non-adherent cells from the supernatant and microscopy (Figure 4a,d). PMA concentrations above 45 ng/mL were intentionally excluded to prevent high toxicity, prolonged oxidative stress, and unwanted baseline inflammatory noise. Concentrations up to 45 ng/mL were sufficient to achieve maximum physical adherence and optimal differentiation efficiency without compromising cell viability or causing artificial cytokine priming [3,4]. Furthermore, flow cytometric analysis revealed that cells exposed to 45 ng/mL PMA exhibited the greatest shifts in scatter profiles while maintaining optimal cell viability and attachment exceeding 90% (Figure 4b–d). Thus, treatment with 45 ng/mL PMA for 48 h, followed by a 24 h resting phase, induced morphological differentiation into resting M0 macrophage-like structures. This optimized protocol was utilized for all subsequent experiments.

To confirm whether the optimized PMA differentiation protocol successfully drives THP-1 cells toward a functionally mature macrophage lineage, morphological changes and macrophage-specific surface markers were evaluated using multi-parameter flow cytometry (Figure 5). As shown by the phase-contrast micrographs (Figure 5a), PMA treatment (45 ng/mL) induced a complete shift from non-adherent, spherical monocytic clusters at assay initiation to firmly attached, spread cells with characteristic macrophage-like morphology following a 24 h resting period. Flow cytometric analysis of forward and side-scatter (FSC/SSC) profiles demonstrated that fully differentiated THP-1 (d-THP-1) cells with characteristic macrophage-like morphology, increased size and cellular complexity, distinguishing the adherent, macrophage-like cells from the undifferentiated monocyte population (Figure 5b). Consistent with these morphological changes, d-THP-1 cells comprised approximately 50% of the total population (Figure 5d), reflecting the inherent phenotypic heterogeneity during PMA-induced differentiation despite high physical adherence (>80%) (Figure 4d). Furthermore, this macrophage-like cell subset exhibited a distinct shift in fluorescence intensity for surface marker expression (Figure 5c). Differentiated THP-1 cells (d-THP-1) exhibited a significant upregulation of the pan-macrophage marker CD68 compared with undifferentiated cells (u-THP-1), confirming successful myeloid maturation [21,22,23]. Additionally, low basal levels of *CD163* expression, a key marker of anti-inflammatory and pro-fibrogenic polarization [21,23], were detected in both undifferentiated and differentiated cells, with no significant difference observed between the two populations. Quantitative analysis (Figure 5d) demonstrated stable expression dynamics across replicates, while revealing distinct phenotypic heterogeneity. Specifically, the protocol yielded approximately 25% CD68-positive and 20% *CD163*-positive populations. These distinct d-THP-1 subpopulations likely represent an M0 baseline and a primary M2-like responder, establishing a platform suitably primed for downstream pro-fibrogenic assays. To verify lineage maturation and baseline polarization state, CD68 was selected as a definitive pan-macrophage marker to confirm successful myeloid differentiation. Concurrently, *CD163* was analyzed as a canonical M2/pro-fibrogenic surface marker to evaluate whether PMA differentiation alone induces pre-existing activation prior to secondary ligand (CXCL12) stimulation. High-dose or prolonged PMA exposure often leads to spontaneous classical (M1-like) activation and persistent baseline expression of inflammatory mediators. Treatment with a moderate PMA dose (45 ng/mL) followed by a 24 h serum-starved resting period allows cells to recover from initial acute PKC activation, minimizing background signaling and resetting the cells to a stable M0 baseline for downstream assays [4].

The chemokine CXCL12 system plays a multifaceted role in modulating macrophage activation, recruitment, and functional plasticity during inflammation and tissue repair [9,24]. To validate the biological sensitivity of our established in vitro model, particularly fibrogenic activity, we stimulated u-THP-1 and d-THP-1 cells with exogenous CXCL12 for 1 h, 3 h, and 24 h and evaluated gene expression dynamics (Figure 6 and Figure 7, respectively). Initially, we examined the expression of the key fibrosis-associated cytokine *TGF-β1* in undifferentiated monocytes following CXCL12 stimulation (Figure 6). Treatment with CXCL12 (10 and 100 ng/mL) failed to induce a sustained fibrosis-associated *TGF-β1* upregulation. After no significant transcriptional changes at 1 h, 10 ng/mL CXCL12 induced a transient elevation at 3 h that returned to baseline by 24 h. Conversely, the higher concentration of 100 ng/mL downregulated *TGF-β1* expression at 3 and 24 h. This minimal and suppressive response suggests a lack of stable receptor coupling or functional exhaustion in suspension cells [25,26]. In contrast, the positive control stimulation with 45 ng/mL PMA maintained significant *TGF-β1* upregulation through 24 h [13,27]. Notably, acute PMA stimulation in u-THP-1 cells induces a substantial upregulation of *TGF-β1* mRNA (Figure 6), indicating cellular transcriptional responsiveness.

Furthermore, the optimized d-THP-1 macrophages, which underwent a 24 h resting phase to recover from initial PMA exposure and establish a stable physiological baseline [3,4], were assessed for their functional response to CXCL12 by assessing the gene expression of pro-fibrogenic (*MMP9* and *TGF-β1)*, M1 proinflammatory (*TNF-α* and *IL-1β*) and M2 (*CD206* and *CD163*) markers. The cells exhibited distinct, gene-specific transcriptional kinetics upon CXCL12 stimulation (Figure 7a–f). Acute CXCL12 stimulation did not induce a sustained pro-inflammatory shift or M1 macrophage phenotype. *TNF-α* mRNA expression remained largely unchanged, showing only a slight downregulation at 3 h with 100 ng/mL CXCL12 (Figure 7a). Similarly, *IL-1β* exhibited a transient upregulation at 3 h induced by 100 ng/mL CXCL12 before rapidly returning to baseline levels by 24 h (Figure 7b). Moreover, CXCL12 treatment did not promote classic M2 marker polarization. While *CD206* expression showed no early activation and a minor decrease at 24 h under 100 ng/mL CXCL12 (Figure 7c), *CD163* expression remained stable at 1 and 3 h but markedly downregulated at 24 h by both 10 and 100 ng/mL CXCL12 (Figure 7d). CXCL12 at 10 ng/mL induced an immediate early upregulation of *MMP9*, but the higher concentration at 100 ng/mL CXCL12 slightly caused *MMP9* upregulation at 1 h, which subsided by 3 h and showed minor suppression at 24 h (Figure 7e). While no significant changes in *TGF-β1* were observed during early time points (1 h and 3 h), treatment with 10 ng/mL CXCL12 induced a statistically significant upregulation of *TGF-β1* at 24 h (Figure 7f).

These transcriptional dynamics suggest that CXCL12 signaling in resting d-THP-1 macrophages triggers a specialized tissue-remodeling and late-stage fibrosis-associated cascade rather than a classical M1 or M2 polarization. The transient early induction of *IL-1β* (3 h) and *MMP9* (1 h) reflects an initial acute phase of cell activation and extracellular matrix remodeling, consistent with CXCL12/CXCR4-mediated signaling in cell migration and matrix degradation [28,29,30]. Furthermore, the late-phase upregulation of *TGF-β1* at 24 h coupled with the downregulation of classic M2 surface markers such as *CD163* and *CD206* potentially indicates that CXCL12 biases macrophage toward a distinct fibrosis-associated signaling axis rather than sustaining an anti-inflammatory phenotype [12,31]. Notably, the lower concentration of CXCL12 (10 ng/mL) elicited stronger transcriptional responses in *MMP9* and *TGF-β1* than the saturating dose (100 ng/mL), demonstrating a characteristic biphasic dose response of chemokine signaling pathways [25,26,32]. Collectively, these findings demonstrate that optimized d-THP-1 macrophages acquire functional receptor coupling to CXCL12 [3,24], driving a controlled transcriptional cascade involving transient early remodeling (*MMP9* and *IL-1β*) and delayed fibrosis-associated priming (*TGF-β1*). While upregulation of *TGF-β1* mRNA indicates transcriptional sensitivity in this model, this pro-fibrogenic transition requires further characterization. Further functional validation, such as total or active *TGF-β1* secretion assays (ELISA), fibroblast co-culture, and SMAD2/3 phosphorylation analysis, is recommended as indicated in recent standardization frameworks [7,8]. Thus, our current findings represent an essential transcriptional priming phase rather than a fully established pro-fibrogenic phenotype. Additionally, to highlight the methodological advantages and functional reliability of our optimized protocol relative to published standards, a direct side-by-side comparison with conventional protocols reported in benchmark studies [3,4,6,21,23] is summarized in Supplementary Table S1. Compared with traditional protocols utilizing high-dose PMA concentrations without a resting phase, our scheme effectively balances high physical cell adherence (>90%) and viability (>80–90%) with minimal baseline inflammatory noise and preserved secondary responsiveness.

## 5. Reagent Setup

PMA Stock Solution (2 mg/mL): Reconstitute 1 mg of PMA powder in 0.5 mL of DMSO. Vortex thoroughly to ensure complete dissolution. Aliquot into sterile, light-protected tubes and store at −20 °C. Avoid repeated freeze–thaw cycles.

PMA Working Solution: Dilute the stock solution in sterile complete RPMI medium to make a 2 μg/mL. Add this directly to complete RPMI medium to reach final 5, 15, or 45 ng/mL immediately before cell seeding.

4% PFA: Dissolve 2 g of PFA in 50 mL of sterile 1× PBS, warm slowly until clear. The solution must be prepared in fume hood. Store at 4 °C.

Human Staining Buffer, 1× (HSB): Mix 500 mL of sterile 1× PBS, 10 mL of human serum group AB, and 1 mL of azide at a final concentration 20% azide. Store at 4 °C.

Recombinant Human CXCL12 Stock Solution (100 μg/mL): Reconstitute 10 μg of recombinant human CXCL12 in water to 100 μg/mL. Aliquot into sterile tubes and store at −20 °C. Avoid repeated freeze–thaw cycles.

Recombinant Human CXCL12 Working Solution: dilute the stock solution in sterile culture medium containing 0.1% FBS.

## 6. Patents

The work reported in this manuscript did not result in any patent applications.

## Figures and Tables

**Figure 3:**
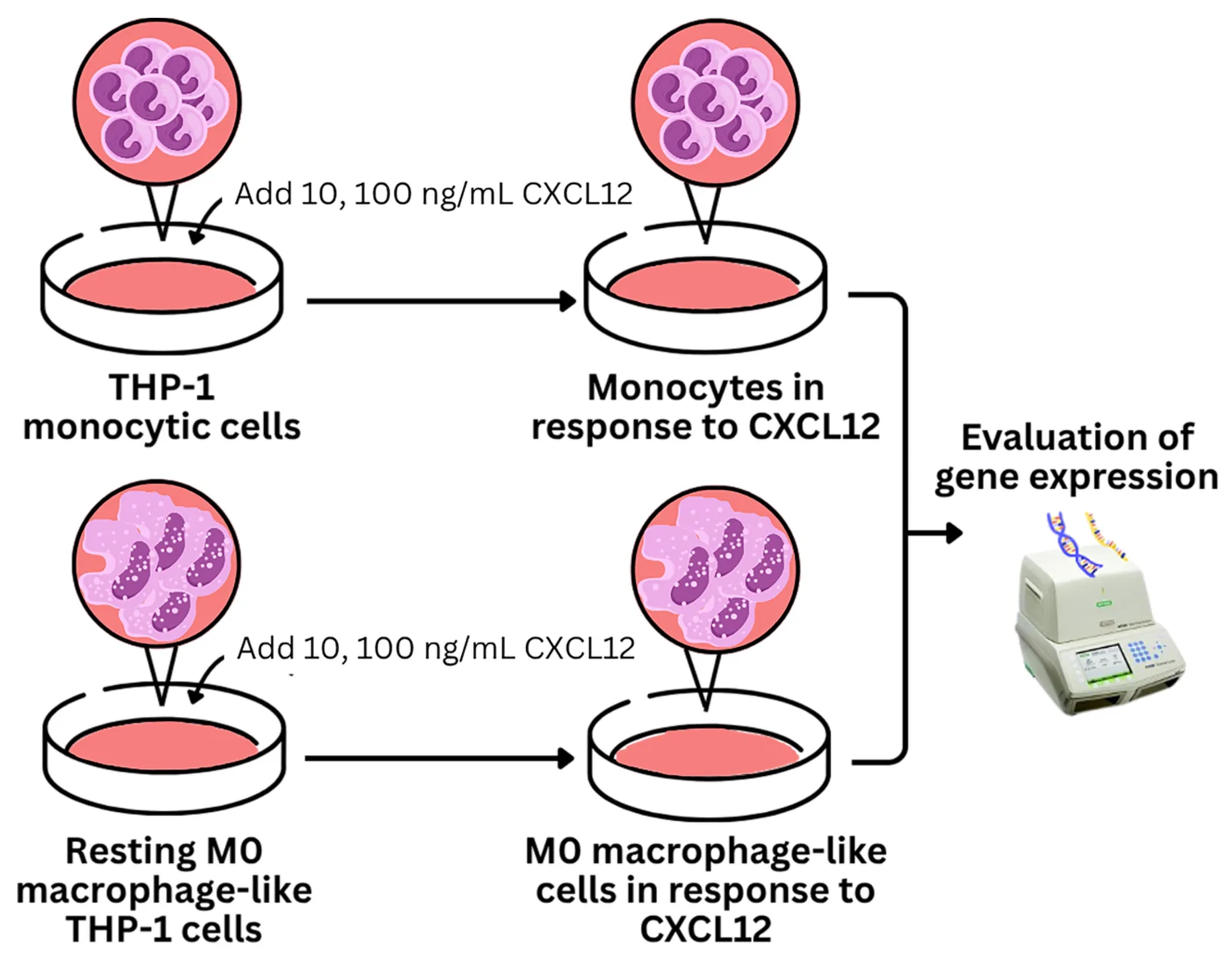
Evaluation of the fibrosis-associated expression potential of CXCL12-stimulated monocytes and macrophage-like THP-1 cells by quantifying gene expression.

**Figure 4:**
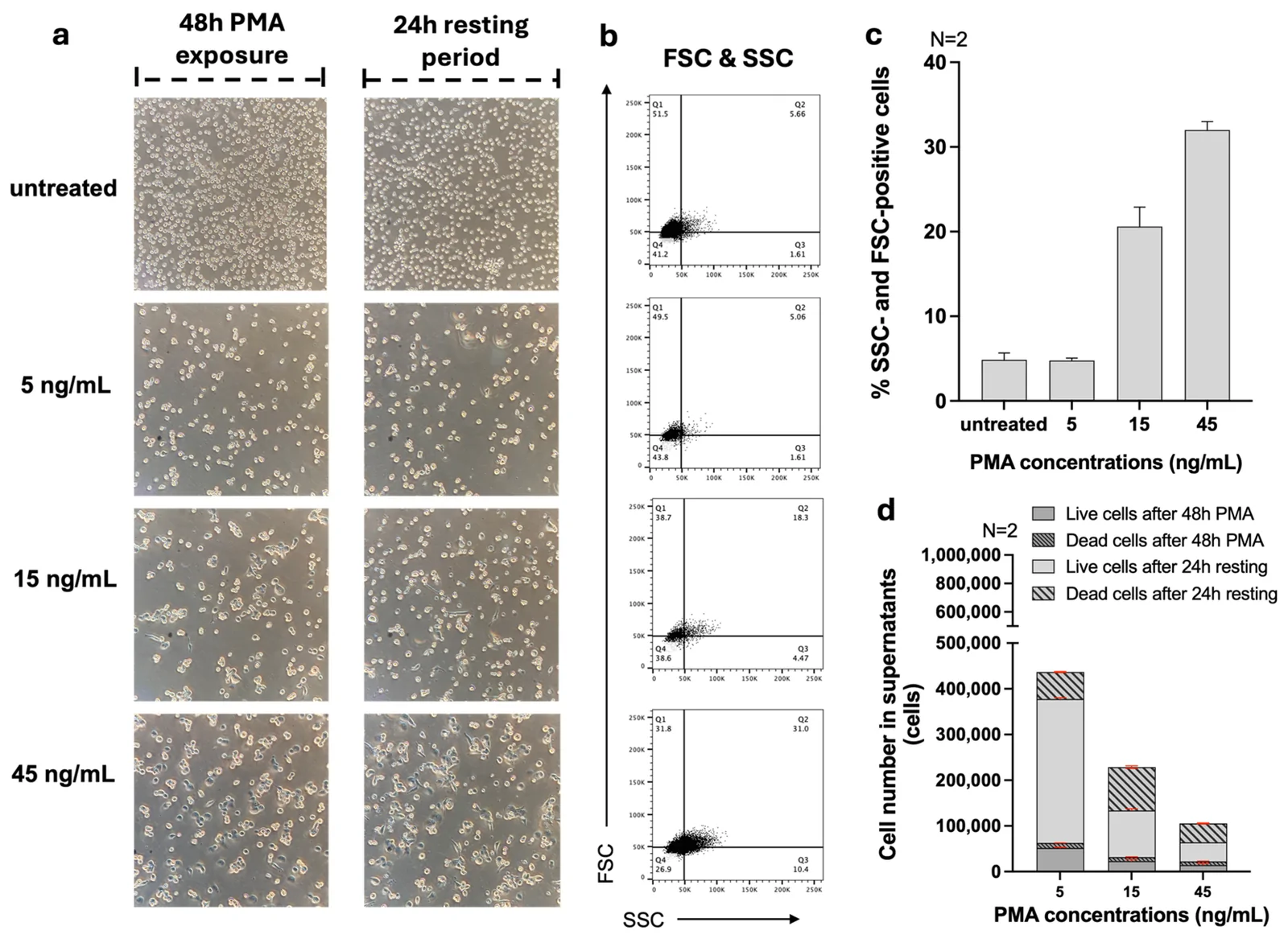
Optimization and characterization of phorbol 12-myristate 13-acetate (PMA)-induced differentiation of THP-1 cells. (**a**) Representative phase-contrast microscopic images demonstrating dose-dependent transition of spherical suspension THP-1 monocytes into adherent, spindle-shaped, macrophage-like cells under a gradient of PMA concentrations (untreated, 5, 15, and 45 ng/mL) at designated incubation time points. Images were captured directly through 6-well plates using an inverted microscope at 10× magnification. (**b**) Flow cytometry dot plots characterizing morphologic shifts in cell size (FSC) and granularity (SSC) after a 24 h resting period. (**c**) Quantitative evaluation of differentiation efficiency, showing the percentage of untreated and differentiated cells at each PMA concentration after a 24 h resting period. (**d**) Non-adherent cell counts, both live and dead, at each step of conditioned medium aspiration; after 48 h PMA exposure and 24 h resting phases, to determine adherence rates. The results were generated from 2 independent experiments. Phorbol 12-myristate 13-acetate, PMA; forward scatter, FSC; side scatter, SSC.

**Figure 5:**
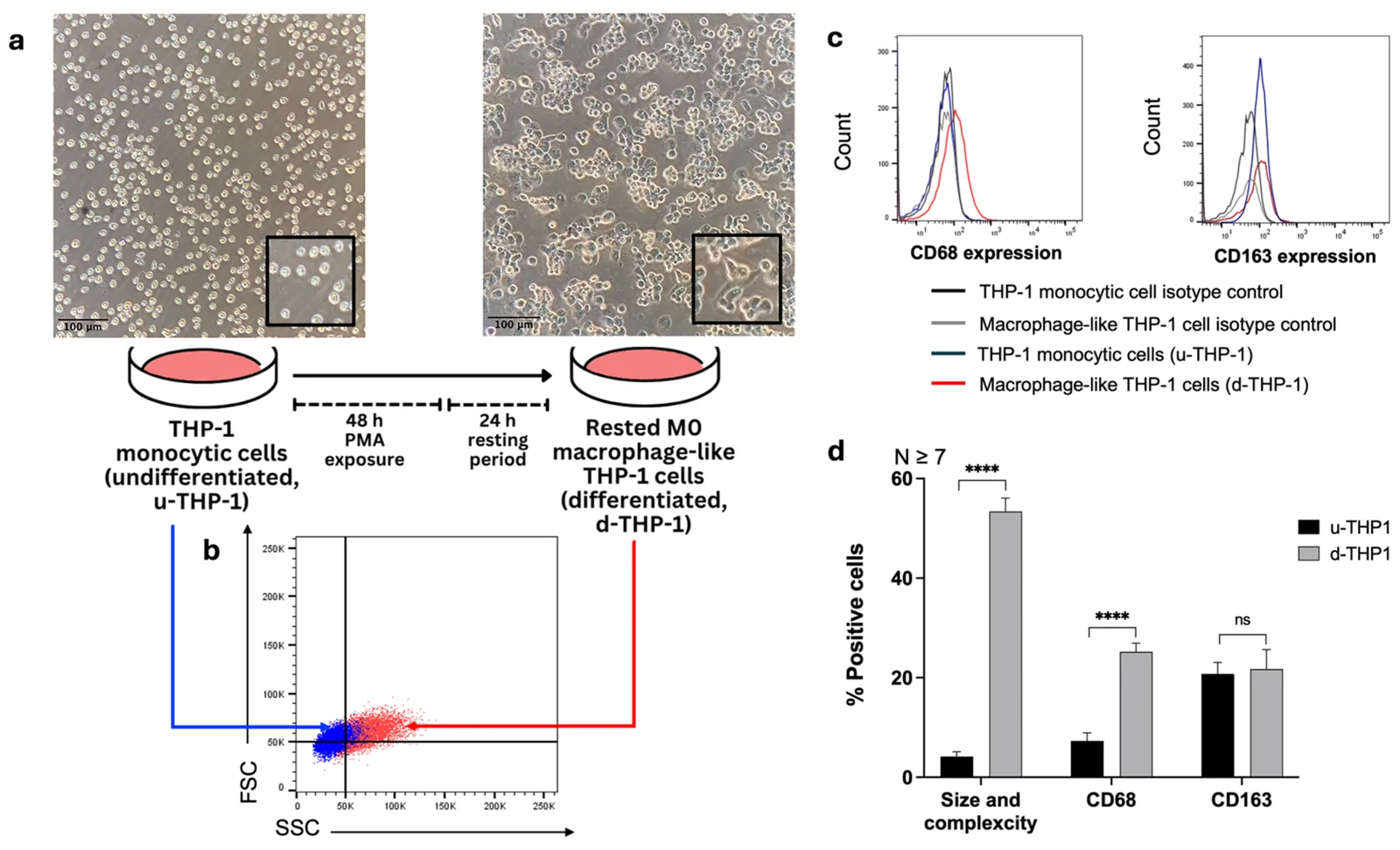
Phenotypic validation and surface marker characterization of macrophage-like THP-1 cells. (**a**) Representative phase-contrast microscopy images illustrating cell morphology before and after treatment with 45 ng/mL PMA at assay initiation and a subsequent 24 h resting period. Images were captured at 10× magnification; scale bar = 100 μm (insets show magnified views of cell morphology). Data are representative of *N* = 4 independent experiments. (**b**) Flow cytometric overlay dot plot demonstrating cell population gating and shift dynamics between untreated monocytes and differentiated macrophage-like cells. (**c**) Representative flow cytometry histograms detailing the mean fluorescence intensity (MFI) and distribution shifts of the pan-macrophage marker CD68 (**left**) and the M2-associated pro-fibrogenic marker *CD163* (**right**). (**d**) Quantitative bar graph summarizing the relative expression levels or percentage of positive cells under specified treatment conditions. Data are presented as the mean ± SEM from independent biological replicates (*N* ≥ 7). Statistical significance between designated groups is denoted by asterisks (ns, no significance, and **** *p* < 0.0001). Representative flow cytometry dot plot showing an overlay of THP-1 cells. Blue dots represent the THP-1 monocytic cells (undifferentiated, u-THP-1), while red dots represent the rested M0 macrophage-like THP-1 cells (differentiated, d-THP-1). Phorbol 12-myristate 13-acetate, PMA; undifferentiated THP-1, u-THP-1; differentiated THP-1, d-THP-1.

**Figure 6:**
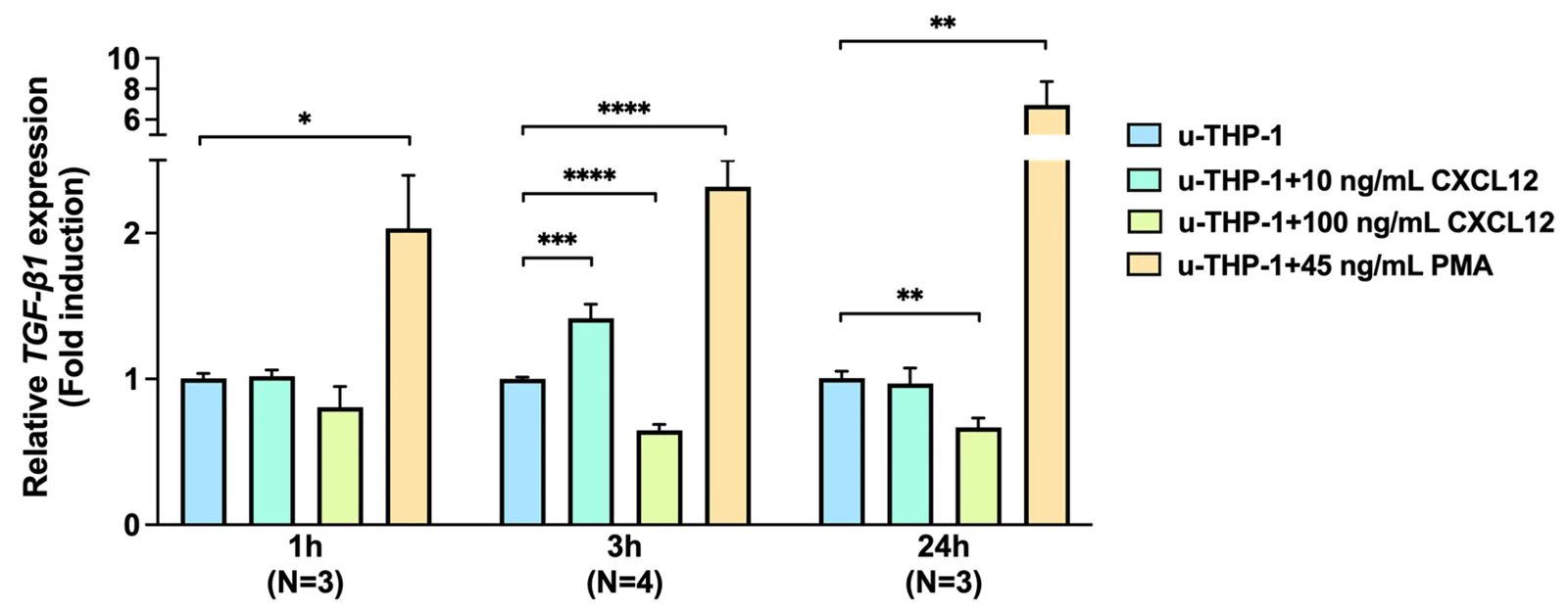
Responsiveness of undifferentiated THP-1 (u-THP-1) cells to CXCL12. Relative *TGF-β1* mRNA expression in u-THP-1 cells at 1 h, 3 h, and 24 h post-treatment with recombinant human CXCL12 (10 or 100 ng/mL) and PMA (45 ng/mL) as a positive control, relative to time-matched untreated baseline. Target gene expression levels were normalized to *GAPDH* as the internal control. Data represent the mean ± SEM of independent replicates (*N* = 3–4 as indicated). Statistical significance between designated groups is denoted by asterisks (* *p* < 0.05, ** *p* < 0.01, *** *p* < 0.001, and **** *p* < 0.0001). Phorbol 12-myristate 13-acetate, PMA; undifferentiated THP-1, u-THP-1; transforming growth factor-beta, *TGF-β1*; C-X-C-motif chemokine ligand 12, CXCL12.

**Figure 7:**
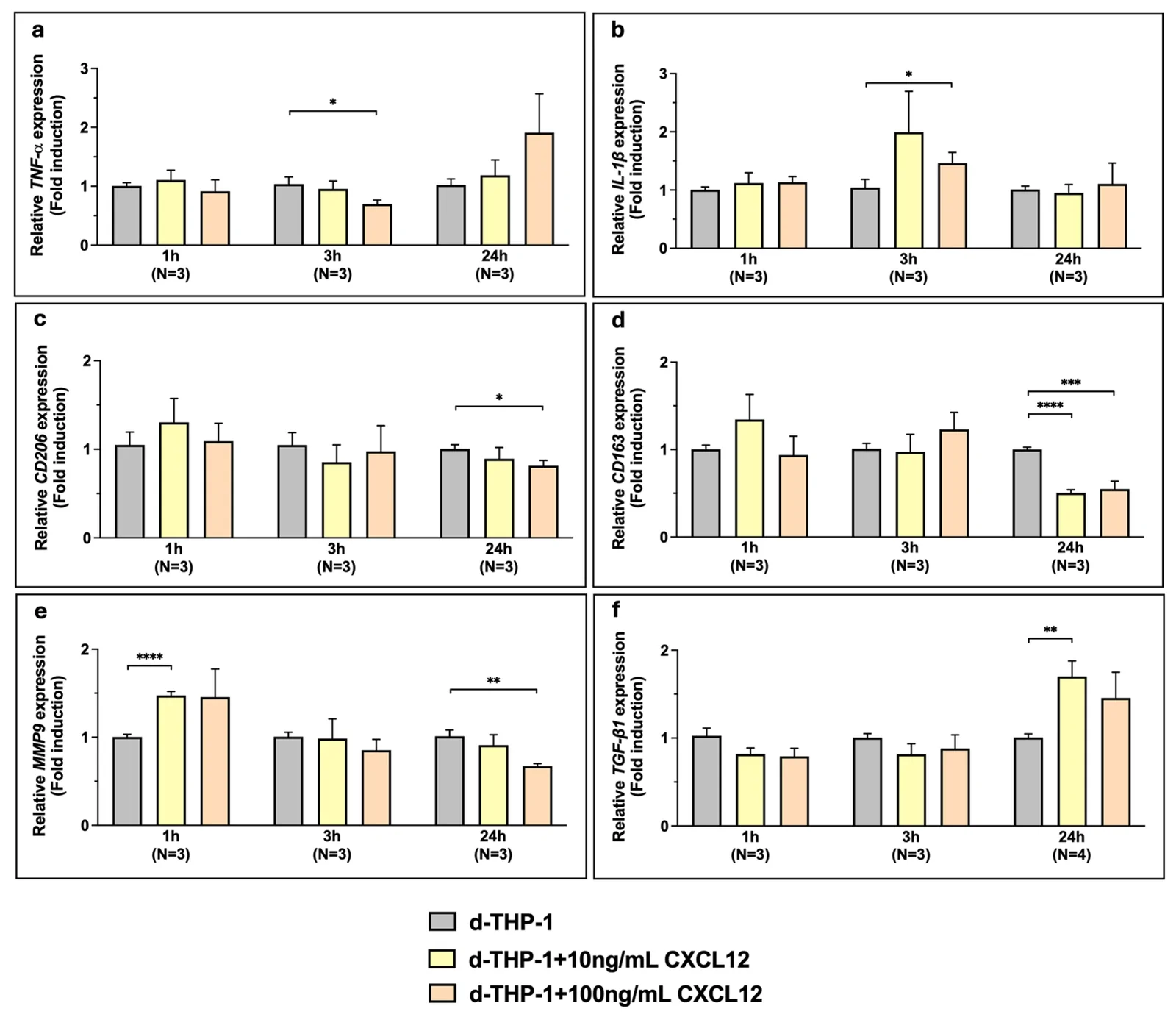
Functional responsiveness of differentiated THP-1 (d-THP-1) cells to CXCL12. Relative (**a**) *TNF-α*, (**b**) *IL-1β*, (**c**) *CD206*, (**d**) *CD163*, (**e**) *MMP9*, and (**f**) *TGF-β1* mRNA expression in d-THP-1 cells at 1 h, 3 h, and 24 h post-stimulation with CXCL12 (10 or 100 ng/mL) relative to time-matched untreated baseline (d-THP-1). Target gene expression levels were normalized to *GAPDH* as the internal control. Data represent the mean ± SEM of independent replicates (*N* = 3–4 as indicated). Statistical significance between designated groups is denoted by asterisks (* *p* < 0.05, ** *p* < 0.01, *** *p* < 0.001, and **** *p* < 0.0001). Differentiated THP-1, d-THP-1; tumor necrosis factor-alpha, TNF-α; interleukin-1 beta, *IL-1β*; cluster of differentiation 206, *CD206*; cluster of differentiation 163, *CD163*; matrix metalloproteinase 9, *MMP9*; transforming growth factor-beta, *TGF-β1*; C-X-C-motif chemokine ligand 12, CXCL12.

## Data Availability

All data generated or analyzed during this study are included within this published article.

## Supplementary Materials

The following supporting information can be downloaded at: https://www.mdpi.com/article/10.3390/mps9050140/s1.

## Abbreviations

The following abbreviations are used in this manuscript:

*CD163*	Cluster of Differentiation 163
*CD206*	Cluster of Differentiation 206
CXCL12	C-X-C Motif Chemokine Ligand 12
d-THP-1	Differentiated THP-1 Cells
*IL-1β*	Interleukin-1 Beta
*MMP9*	Matrix Metalloproteinase 9
PMA	Phorbol 12-Myristate 13-Acetate
RT-qPCR	Reverse-Transcription Quantitative Polymerase Chain Reaction
*TGF-β*	Transforming Growth Factor-Beta
TNF-α	Tumor Necrosis Factor-Alpha
u-THP-1	Undifferentiated THP-1 Cells
