# Supplementary material for: An Optimized Protocol for the Differentiation and Fibrosis-Associated Transcriptional Priming of THP-1 Macrophages
Source: Methods Protoc. 2026 Sep 25;9(5):140. doi: 10.3390/mps9050140 (PMC13707598; doi:10.3390/mps9050140)
Supplement: Supplementary file 1 [file mps-09-00140-s001.zip › mps-4479589-supplementary.pdf]

Table S1 Methodological comparison between conventional protocols and the presented optimized protocol. Literature-based baseline characteristics were retrieved from benchmark studies [3, 4, 6, 21, 23] (Daigneault *et al*., 2010, Lund *et al*., 2016, Maess *et al*., 2014, Genin *et al*., 2015 and Furgiuele *et al*., 2022) to contextualize the functional advantage of the present protocol.

Experimental parameter	Conventional protocols	Optimized protocol (present study)	Biological impact and rationale
PMA concentration and duration	High dose (100-200 nM or ~60-120 ng/mL) for 48-72 h with or without resting	Moderate dose (45 ng/mL or ~73 nM) for 48 h followed by 24-h resting phase	Reduces acute PKC-mediated cellular stress and cytotoxicity
Cell adherence and viability	High doses result in high physical adherence but also high cytotoxicity or apoptosis	High physical adherence (>90%) and optimized cell viability (>80-90%) with minimal cell loss	Preserves monolayer integrity for downstream assays
Baseline inflammatory noise	Sustained baseline expression of pro-inflammatory cytokines (e.g., *TNF-a, IL-1b, IL-8*)	Resets to a stable baseline M0 state with minimal background transcriptional noise	Prevents false-positive activation and background interference
Surface marker profile	High CD68 expression with potential pre-existing classical (M1-like) surface marker activation	CD68 positivity (~25%) and low basal CD163 (~20%) reflecting unprimed M0/M2-like responders	Establishes a clear baseline primed for secondary polarization
Secondary functional responsiveness	Blunted or exhausted intracellular signaling pathways upon secondary ligand stimulation	Preserved signaling integrity biphasic upregulation of *TGF-β1* and *MMP9* mRNA upon CXCL12 stimulation	Confirms functional receptor coupling and transcriptional readiness
